# Pulsed Radiofrequency in the Management of Postsurgical Abdominal Wall Chronic Pain: A Report From a Single Oncological Center

**DOI:** 10.7759/cureus.67136

**Published:** 2024-08-18

**Authors:** Sofia Trovisco, Gonçalo Bem, Manuel Silva, Ana Agrelo

**Affiliations:** 1 Department of Anesthesiology and Intensive Care Medicine, Instituto Português de Oncologia do Porto Francisco Gentil EPE, Porto, PRT; 2 Department of Anesthesiology and Intensive Care Medicine, Unidade Local de Saúde de São João EPE, Porto, PRT

**Keywords:** neuropathic pain treatment, neuromodulation, chronic postsurgical pain, inguinal neuralgia, meralgia paraesthetica, pulsed radiofrequency

## Abstract

Chronic postsurgical pain (CPSP) is defined as pain that develops or increases in intensity after a surgical procedure or tissue injury and persists beyond the healing process, lasting at least three months after the precipitating event. Often neuropathic in nature, CPSP can be challenging to manage. CPSP is a common complication, with data suggesting an incidence ranging from 5% to 85%, depending on the type of procedure. Meralgia paresthetica (MP) and ilioinguinal/iliohypogastric neuralgias (IH/IL N) are two possible clinical scenarios of CPSP following lower abdominal procedures. Pulsed radiofrequency (PRF) is a minimally invasive technique of peripheral neuromodulation effective in various pain etiologies; however, evidence is scarce regarding its use in MP and IH/IL N. This case series aims to assess the potential role of PRF in the management of CPSP following abdominal wall procedures. This case series was set in a single oncological center between January 2017 and February 2022 and included adult patients (>18 years old) referred to our unit with a high suspicion of postsurgical MP or IH/IL N refractory to conservative treatment. PRF was performed after a positive diagnostic block in patients whose pain could not be controlled despite optimal medical treatment. The efficacy of PRF was assessed regarding pain intensity using the verbal numeric scale (VNS) and the duration of pain relief in weeks. The follow-up period was from the initial PRF procedure to the end of data collection. Parametric data were presented as mean and standard deviation (SD), and non-parametric data as median (minimum-maximum). Seventeen patients were included: 82.35% (n=14) were female, and the mean age was 58.0 ± 11.35 years. MP was present in 47.1% (n=8) and IH/IL N in 52.9% (n=9). Transverse rectus abdominis muscle flap reconstruction (TRAM) was the most common procedure (n=5, 29.41%). Diagnostic blocks were performed in 88.24% (n=15) of the patients. Initial VNS scores were 7.59 ± 2.62; 2.82 ± 2.62 at 24 hours; and 2.47 ± 1.58 at 15 days. During follow-up, 70.59% (n=12) of patients had no recurrence of initial symptoms. A second PRF was performed in 29.41% (n=5) cases based on the recurrence of symptoms, following a mean period of pain relief of 112 (8-238) weeks. No major or minor complications were identified during early or late follow-up. PRF can be a useful tool to improve the multimodal management of postsurgical abdominal wall chronic pain.

## Introduction

Chronic postsurgical pain (CPSP) is defined as pain that develops or increases in intensity after a surgical procedure or tissue injury and persists beyond the healing process, lasting at least three months after the precipitating event [1]. Data suggest an incidence, depending on the type of operation, ranging from 5% to 85%, with CPSP being more common after amputation, inguinal hernia repair, mastectomy, and thoracotomy [1-4]. The mean incidence following hernia repair surgery is 11% [5], with CPSP typically located either at the surgical area of injury or in its referred area [1]. Other postsurgical causes of pain, such as infection, fluid collection, displacement of surgical material, and cancer recurrence/persistence, should be ruled out [4, 6-9]. CPSP can be nociceptive or neuropathic in nature, the latter being particularly difficult to treat. To establish a diagnosis of neuropathic pain (NP), a history of relevant injury or disease to the somatosensory nervous system and a pain distribution neuroanatomically plausible on neurological examination must be present [2]. NP may paradoxically lead to both a loss of function and increased pain sensitivity and spontaneous pain [2]. The causes of postsurgical NP include nerve entrapment by mesh, sutures, or fibrosis, and neuroma formation associated with partial or complete transection of the involved nerve [10].

Meralgia paresthetica (MP) and ilioinguinal/iliohypogastric neuralgias (IH/IL N) are peripheral neuropathies often considered iatrogenic complications of certain abdominal and perineal surgical procedures [10-18]. Diagnosing these CPSP syndromes is often challenging. A relevant history of nerve injury, an accurate neurological examination, and alleviation of the pain by a diagnostic nerve block are key to diagnosis.

There are several treatment options for CPSP. Conservative pharmacological therapy successfully relieves symptoms in most patients [9, 12, 18]. In refractory cases, spinal cord stimulators, transcutaneous electrical nerve stimulation, and neurectomy have all been tried for pain management [18]. Pulsed radiofrequency (PRF) is a local neuromodulation technique that avoids neuritis and has recently been suggested as an alternative method for CPSP management. PRF is a minimally invasive technique that involves the local application of pulses of low-energy electric current in rapid pulsations and controlled temperature (42 ºC) to target nervous tissue and associated microglia [19]. It has been effective in treating various chronic pain conditions, such as thoracic postherpetic neuralgia, trigeminal neuralgia, radicular pain, occipital neuralgia, and shoulder and knee pain [19]. Although PRF has demonstrated efficacy and has been widely adopted in the treatment of NP, there is sparse literature focusing on the management of postsurgical abdominal wall chronic pain. The purpose of this case series is to further assess the efficacy of PRF in the management of MP and IH/IL N.

## Materials and methods

Study design and settings

This study was conducted at the Department of Anaesthesiology and Intensive Medicine, Portuguese Institute of Oncology of Porto Francisco Gentil EPE, Porto, Portugal, between January 2017 and February 2022 (62 months). We included adult patients (>18 years old) referred to our unit with a high suspicion of postsurgical MP or IH/IL N refractory to conservative treatment. Electronic medical databases were reviewed to identify these patients. Patients with incomplete medical records were excluded. Variables included age, gender, type of prior surgery, type of postsurgical chronic pain, presence of a diagnostic nerve block, and pain intensity scores (verbal numeric scale (VNS) score) before and after procedures.

Pain intensity was evaluated by the same team at different periods: at presentation, on day 1, and on day 15 following PRF. Follow-up was based on clinical outcomes, such as recurrence of pain, between the initial PRF and the end of data collection. During follow-up, the number of PRF procedures for each patient and the duration of pain relief (in weeks) were recorded. Informed consent was obtained. We also assessed possible complications after the procedure. Parametric data are presented as mean and standard deviation (SD). Nonparametric data are presented as median. Statistical analysis was performed using MS Excel 2013 (Microsoft Corporation, Redmond, Washington).

MP and IH/IL N diagnosis

All patients referred to our unit with a high suspicion of refractory postsurgical MP or IH/IL N underwent a thorough anamnesis (verbal descriptions of NP) and physical evaluation (pain distribution neuroanatomically plausible) to assess the presence of a history of relevant neurological damage and Neuropathic Pain 4 Questions (DN4) score > 4. In all cases, multimodal pharmacological therapy was optimized in combination with behavioral interventions. An ultrasound-guided diagnostic block with 5 mL of 0.2% ropivacaine and 40 mg of methylprednisolone was performed. Temporary pain relief >50% was considered a positive response to the diagnostic nerve block, and in these cases, PRF was proposed, and informed consent was obtained.

PRF procedure

For MP PRF, using a high-frequency ultrasound (US) probe, the ASIS was visualized as a hyperechoic structure with posterior acoustic shadowing. The US probe was placed over the ASIS initially with the long-axis view of the inguinal ligament and then moved distally. The sartorius muscle was identified as an inverted triangular structure. The LFCN appeared as one or more hyperechoic or hypoechoic structures in the short-axis view. If the nerve could not be identified, the alternative was to look for the LFCN in the plane between the tensor fascia lata and the sartorius muscle. Once the LFCN was identified, a needle was advanced in-plane with the US probe.

For IH/IN PRF, a linear high-frequency US probe was placed medially and inferior to the ASIS. The II/IH nerves were identified between the internal oblique and transversus abdominis muscles. Selective stimulation of sensory fibers (50 Hz) showing concordant pain or dysesthesia below 0.3 V confirmed proper localization of the PRF electrode. Motor stimulation to exclude the presence of nerves with motor function was negative up to 1.5 V and 2 Hz. The sensory stimulation results were the most important findings used for target selection. After stimulation, PRF neuromodulation was performed as a pulse at a fixed 45 V for four cycles of 120 seconds.

## Results

Between January 2017 and February 2022, 20 patients were identified; three patients were excluded due to incomplete medical records. Of the 17 patients included, 82.35% (n=14) were female (Table 1). The median age was 58.00 ± 11.35 years. MP was present in 47.10% (n=8) of the cases, and IH/II N was present in 52.90% (n=9) (Table 1). Regarding previous abdominal wall surgery, transverse rectus abdominis muscle flap reconstruction (TRAM) was the most prevalent procedure (n=5, 29.41%) (Table 1). All patients underwent PRF following a positive diagnostic nerve block (Table 1). At presentation to our unit, the mean VNS score was 7.59 ± 2.62. The mean VNS scores were 2.82 ± 2.62 on day 1 and 2.47 ± 1.58 on day 15 following the initial PRF procedure, respectively (Table 2). During follow-up, 70.59% (n=12) of patients had no recurrence of initial symptoms, still presenting satisfactory pain control with no need for further PRF. A second PRF, based on the recurrence of symptoms, was performed in five cases. In these, the mean presentation VNS score at the second PRF was 7.40 ± 2.62 (Table 2). On day 1 and day 15 following the procedure, the mean VNS scores were 2.61 ± 2.42 and 3.6 ± 1.02, respectively (Table 2). A third PRF was performed in two cases, with a mean pain VAS score of 7.0 prior to the procedure. On day 1 and day 15 following the procedure, the mean VNS scores were 1.0 ± 1.41 and 3.5 ± 0.52, respectively (Table 2). No major or minor complications were identified.

**Table 1:** Patients’ demographic data, previous surgery, and diagnostic nerve blocks MP: Meralgia paresthetica, IH/IL: Ilioinguinal and iliohypogastric neuralgia, SD: standard deviation, TRAM: transverse rectus abdominis muscle flap reconstruction, IL: Ilioinguinal, IH: iliohypogastric, LCFN: lateral femoral cutaneous nerve, TAP: transversus abdominis plane block.

Variables	MP	IH/IL	Total
Patients, n (%)	8 (47.06)	9 (52.94)	17
Age, mean (SD)	59 (13.27)	59 (11.70)	58 (11.35)
Female, n (%)	8 (100.00)	6 (66.67)	14 (82.35)
Previous surgery			
Lower abdomen/pelvic surgery, n (%)	4 (50.00)	5 (55.56)	9 (52.94)
Inguinal/upper thigh surgery, n (%)	4 (50.00)	4 (44.44)	8 (47.06)
Diagnostic nerve block			
IL + IH, n (%)	0 (0.00)	5 (55.56)	5 (29.41)
LCFN, n (%)	7 (87.50)	1 (11.11)	7 (41.18)
TAP, n (%)	1 (12.50)	2 (22.22)	3 (17.65)

**Table 2:** Pulsed radiofrequency MP: Meralgia paresthetica, IH/IL: ilioinguinal and iliohypogastric neuralgia, PRF: pulsed radiofrequency, VAS: visual analog scale, VNS: verbal numeric scale, NA: not applicable.

Variables	MP	IH/IL	Total
Number of PRF			
1, n (%)	8 (100.00)	9 (100.00)	17 (100.00)
2, n (%)	3 (37.5)	2 (22.22)	5 (29.41)
3, n (%)	0 (0.00)	2 (22.22)	2 (11.76)
4, n (%)	0 (0.00)	1 (11.11)	1 (5.88)
5, n (%)	0 (0.00)	1 (11.11)	1 (5.88)
First PRF			
Pain before, VNS, mean (SD)	7.85 (1.14)	7.59 (1.50)	7.59 (2.62)
Pain 24h after, VNS, mean (SD)	2.62 (2.69)	2.82 (2.70)	2.82 (2.62)
Pain 15 days after, VNS, mean (SD)	2.46 (1.56)	2.47 (1.62)	2.47 (1.58)
Time of relief (weeks), median (min-max)	152 (18-236)	112 (8-238)	112 (8-238)
Second PRF			
Pain before, VNS, mean (SD)	7.33 (1.53)	7.4 (1.14)	7.4 (1.02)
Pain 24h after, VNS, mean (SD)	1.67 (1.15)	2.6 (2.7)	2.6 (2.42)
Pain 15 days after, VNS, mean (SD)	4.33 (0.58)	3.6 (1.14)	3.6 (1.02)
Time of relief (weeks), median (min-max)	43 (36-56)	36 (10-24)	36 (10-56)
Third PRF			
Pain before, VNS, mean (SD)	NA	7 (0.00)	7 (0.00)
Pain 24h after, VNS, mean (SD)	NA	1 (1.41)	1 (1.41)
Pain 15 days after, VNS, mean (SD)	NA	3.5 (0.71)	3.5 (0.71)
Time of relief (weeks), median (min-max)	NA	26 (8-44)	26 (8-44)
Fourth PRF			
Pain before, VNS, mean (SD)	NA	8	8
Pain 24h after, VNS, mean (SD)	NA	0	0
Pain 15 days after, VNS, mean (SD)	NA	0	0
Time of relief (weeks), median (min-max)	NA	24	24
Fifth PRF			
Pain before, VNS, mean (SD)	NA	8	8
Pain 24h after, VNS, mean (SD)	NA	3	3
Pain 15 days after, VNS, mean (SD)	NA	6	6
Time of relief (weeks), median (min-max)	NA	8	8

## Discussion

MP and IH/IL N are peripheral neuropathies often considered iatrogenic complications of certain abdominal and perineal surgical procedures [10, 11]. Diagnosing these CPSP syndromes is often challenging. A relevant history of nerve injury, an accurate neurological examination, and alleviation of pain by a diagnostic nerve block are key to diagnosis. Imaging and neurophysiological modalities can be useful to confirm the diagnosis in ambiguous cases [7-9, 12]. MP was first described by Martin Bernhardt in 1878, and in 1885, Hager postulated that lateral femoral cutaneous nerve (LFCN) injury is the cause of the pain. MP is a sensory mononeuropathy of multifactorial etiology with a reported incidence of about 4.3/10,000 [13, 14], and an incidence of 0.82% following gynecological oncological surgery [15]. It is generally caused by the entrapment of the LFCN, a purely sensory nerve branch derived from the posterior divisions of the anterior rami of the L2 and L3 spinal nerves. As the nerve travels down the pelvis and lateral to the psoas muscle, it crosses the iliacus before passing beneath the inguinal ligament [7]. It then traverses medial to the anterior superior iliac spine (ASIS), ultimately piercing the fascia lata before giving rise to its terminal cutaneous branches [7]. Symptoms vary from paresthesia and numbness to burning discomfort along the distribution of the LFCN. Diagnosis of MP is confirmed by either nerve conduction studies or a diagnostic nerve block to the LFCN. Conservative pharmacological management provides satisfactory results in around 85% of MP patients [13]. Refractory cases have been treated with various other interventional procedures, such as local anesthetic (LA) or alcohol infiltration around the LFCN, PRF of the LFCN, and surgical treatment, including decompression and transection of the nerve [13]. IH/IL N is an entrapment syndrome hypothesized to result from mechanical compression of the IH/IL nerves. IH/IL N is an underestimated cause of CPSP. After major pelvic surgery, obturator and IH/IL nerve injuries are the most common postoperative neuropathies. The incidence of these underdiagnosed neuropathies after gynecological surgery or a Pfannenstiel incision has been reported to be 3-7% [10, 16]. The disorder is characterized by sharp, shooting pain originating at the ASIS and radiating to the groin. Anatomically, the entrapment is thought to occur as the nerves pass deep to the transverse abdominis muscle, just medial to the ASIS. IH/IL N in postoperative patients may be secondary to fibrous adhesions, neuroma formation, or suture placement. Diagnosis of IH/IL N is difficult and based on history, physical examination, and alleviation of pain by a diagnostic nerve block. It is challenging to discriminate between IH and IL neuropathies because of their common origin (T12 and L1 nerve roots) and overlapping distribution [10]. They descend on the quadratus lumborum muscle along the parietal peritoneum, perforate the transverse abdominal muscle, and course between the transverse abdominal muscle on the inside and the lesser oblique on the outside at the level of the ASIS. Both the IH/IL nerves then pass along the inguinal canal to become subcutaneous in their territories of distribution [17]. In contrast with the IL nerve, the cutaneous distribution of the IH nerve does not usually extend into the groin or scrotum.

Neuropathic pain, especially chronic neuropathic pain after peripheral nerve injury, is often challenging, particularly in the oncological setting. Etiology-specific techniques involving regional analgesia are now being used more consistently [20].

Conventional radiofrequency (CRF) has proven efficacy in refractory pain, including IH/IL N [17]. It uses temperature to produce a thermal lesion in a target nerve, resulting in the interruption of nociceptive afferent pathways [21]. However, the high temperatures used can cause neurolysis and irreversible loss of function, with the potential to cause neuritis-like reactions associated with neuropathic pain [21]. Pain physicians should, therefore, consider temporary but possibly long-lasting alternatives to CRF, such as PRF.

PRF is a minimally invasive technique found to have clinical utility in managing chronic neuropathic syndromes. It involves the application of pulses of electric current to achieve pain control through local neuromodulation, while avoiding neuritis-like reactions, motor dysfunction, and deafferentation pain. This approach results in longer pain relief compared to infiltrations with LA and corticosteroid [17, 22]. Nevertheless, the mechanism by which PRF causes pain relief remains debatable [19].

Our study includes a total of 17 oncological patients with postsurgical abdominal neuropathy refractory to medical treatment who were successfully managed with PRF. We achieved a satisfactory reduction in pain levels, with a decrease from a mean VNS score of 7.59 before the procedure to 2.82 after 24 hours, with both groups (MP and IH/IL N) showing similar results. These pain reductions are consistent with those reported in the literature. In a previously published case series [23], the authors described a reduction in MP pain in five patients following PRF, with a decrease in VNS from 8.0 to 2.9. The experience with PRF in IH/IL N seems to be lacking, with only a few case reports describing similar reductions after PRF [5, 16, 24].

We achieved a mean period of pain relief of 152 weeks (35 months) for MP and 112 weeks (26 months) for IH/IL N after a single PRF. Previous studies have reported shorter follow-up times of three months to two years for MP [7, 11, 13, 14, 18, 23, 25-27] and 3 to 9 months for IH/IL N [5, 22, 24]. Lee et al. reported a retrospective observation of 11 patients with MP who underwent PRF [25]. All patients experienced significant pain relief (VAS, ≥50% reduction in pain) during the six-month follow-up. At the end of the follow-up, seven patients were pain-free, three had successful pain relief (≥50% reduction in pain as determined by VAS scores), and one had recurrence of pain. The average follow-up time was 15 months. Makharita et al. conducted a randomized, double-blind controlled trial in which 21 patients with chronic inguinal neuralgia were allocated to PRF (two cycles of 120 seconds each) in the DRG of T12, L1, and L2 nerve roots or a sham procedure, with both groups receiving LA and corticosteroid [22]. PRF provided significantly longer-lasting pain relief compared to LA- and steroid-selective nerve root blockade, with a significantly longer duration (17.7 ± 3.9 weeks versus 7.6 ± 1.6 weeks).

In 71% of patients, we were able to provide major and sustained pain reduction with a single PRF. In three MP patients, PRF was repeated a second time, as the pain, although with less intensity, relapsed. These three patients achieved satisfactory pain control for a period ranging from 36 to 56 weeks after the second PRF. Carola et al. reported a successful single case in which PRF was repeated three weeks after the first procedure to further reduce pain intensity [14]. Regarding IH/IL N, two patients required more than one PRF, with one patient requiring three PRFs and the other five. In the latter, PRF achieved significant pain reduction lasting from 8 to 24 weeks, after which the pain resurfaced. Unlike PRF for the LFCN, the need for repeated PRF for IH/IL N pain control seems to be more common. Makharita et al. reported that three to four PRF blocks were needed to achieve long-lasting pain relief [22].

Traditionally, PRF in MP uses 42ºC stimulation for 120 seconds [13, 14, 25, 26], with variable rates of pain reduction and pain relief. However, it was recently demonstrated that long-lasting PRF (six to eight minutes) for the trigeminal and saphenous nerves was associated with longer pain relief and greater patient satisfaction [28-30]. This approach in MP is relatively recent, first described in 2018, although it has been used for other pathologies [23]. Regarding IH/IL N, studies found in the literature mostly performed two-minute PRF, except for Alici et al. and Thapa et al., who used six and eight minutes, respectively [5, 16]. In our study, we applied extended PRF (eight minutes), resulting in pain relief times ranging from 8 to 238 weeks. We believe that extending PRF stimulation time allows for superior and more consistent pain reduction and contributes to a longer period of pain relief.

Several PRF techniques have been proposed, ranging from landmark-based to imaging-guided techniques, with the latter associated with better pain relief scores. In our study, PRF was performed targeting the LFCN or IH/IL nerves through US guidance as previously described [5, 13, 16, 23]. We believe this is the best option as it allows real-time visualization of anatomical structures and confirmation of precise needle location immediately adjacent to the nerve. Other advantages include a more rapid procedure, logistical ease (no need for an operating room), and the avoidance of radiation exposure required in CT and fluoroscopy. At the same time, the complication rate does not seem to be higher compared to these two options.

This study has some limitations. It was a retrospective case series involving a small sample of patients. Additionally, the outcome measure was determined using only a pain assessment scale. However, it provides a promising view of the effectiveness and safety of PRF in these patients.

## Conclusions

This study shows promising results regarding the use of PRF in the management of MP and IH/IL N refractory to medical therapies, with reports of VNS scores <3 following PRF. Although further research is needed, our median period of pain relief of 28 months is encouraging, with no complications reported. Extending PRF time to eight minutes does not appear to cause any side effects, while it seems to increase pain relief duration. Larger prospective studies should be conducted to properly evaluate the most effective PRF duration and identify which patients would be the most appropriate candidates for this procedure.
